# Study on Rheological Properties and Enhancement Mechanisms of Ethylene-Vinyl-Acetate-Copolymer-Modified Cement Grouting Materials

**DOI:** 10.3390/ma19050965

**Published:** 2026-03-02

**Authors:** Jiehao Wu, Nianzu Zhang, Duoxi Yao, Yuxuan Wang

**Affiliations:** 1 School of Civil Engineering and Architecture, Anhui University of Science and Technology, Huainan 232001, China; 2021096@aust.edu.cn (J.W.); zhangnianzu1225@foxmail.com (N.Z.); 2 National-Local Joint Engineering Laboratory of Building Health Monitoring and Disaster Prevention Technology, Hefei 230601, China; 3 School of Earth and Environment, Anhui University of Science and Technology, Huainan 232001, China; dxyao@aust.edu.cn; 4 State Key Laboratory of Safe Mining of Deep Coal and Environmental Protection, Anhui University of Science and Technology, Huainan 232001, China

**Keywords:** ethylene-vinyl acetate, cement-based grouting materials, microstructure, mechanical enhancement, rheological behavior, grouting reinforcement

## Abstract

This study addresses the brittleness, poor bonding, and low crack resistance of ordinary Portland cement (OPC) grouting materials by incorporating an ethylene-vinyl acetate (EVA) copolymer. The enhancement mechanisms and engineering applicability of EVA-modified cement grouts were systematically investigated. Using EVA contents from 0% to 20%, macro-scale tests covering fluidity, rheology, bleeding rate, and compressive strength were conducted, along with microstructural analyses (SEM, XRD, FT-IR). Results indicate that with 12% EVA, the 28-day compressive strength reached 21.03 MPa, reflecting a 68% increase over the unmodified grout. Most favorable amount of EVA promoted the formation of C–S–H gel, filled microcracks, and enhanced structural densification, whereas excessive EVA content led to the formation of a polymer film that hindered hydration and reduced strength. Furthermore, EVA effectively improved the rheological behavior of the grout, with the Vipulanandan model demonstrating superior accuracy over the Bingham model in characterizing its non-Newtonian flow. This study systematically established a quantitative–qualitative correlation between EVA content, nonlinear rheological behavior (characterized by advanced models), microstructure evolution (porosity, C–S–H, polymer film) and final macromechanics and durability.

## 1. Introduction

The stability of surrounding rock constitutes a fundamental challenge in underground engineering, particularly in coal mining [1,2,3]. Grouting reinforcement, an effective method to enhance the bearing capacity of fractured rock masses and control deformation, is widely employed [4,5]. The choice of grouting material is decisive for the reinforcement outcome. Currently, ordinary Portland cement (OPC) remains the most prevalent material due to its accessibility and cost-effectiveness [6,7,8]. However, traditional grouting materials based on OPC, despite their advantages of high strength and wide availability, suffer from inherent drawbacks such as high brittleness, poor bonding performance, and susceptibility to shrinkage cracking. These limitations severely compromise the integrity and long-term durability of reinforced structures, thereby restricting their effectiveness in critical engineering applications. Additionally, the presence of calcium hydroxide and aluminate phases in the hydration products further reduces their resistance to soft water erosion and chemical corrosion [9,10].

In recent years, significant efforts have been devoted to developing advanced grouting materials. Gullu [11] studied the use of sludge ash as an additive to polypropylene fiber (PF), finding that it notably improved the unconfined compressive strength (UCS) of sand. Adding polypropylene fiber further enhanced the stress–strain behavior and reduced post-peak strength loss. Fan et al. [12] applied grouting to seal water-conducting channels in Ordovician limestone aquifers, effectively mitigating water inrush risks in mining faces. Liu et al. [13] compared the reinforcement performance of sulfoaluminate cement, OPC, and epoxy resin on fractured rock, noting that polymer-based materials offered superior strength and deformation properties. Zhang et al. [14] created a fly ash-based grouting material toughened by in situ acrylamide polymerization, which exhibited high toughness and water-swelling capacity, making it suitable for controlling water seepage in mine roofs.

Among various polymer modifiers, EVA demonstrates a unique application potential [15,16]. Compared with other polymers, the vinyl acetate segment in the EVA molecular chain may be hydrolyzed in an alkaline environment to form a carboxyl group. These carboxyl groups can interact with calcium ions in the hydration products, which helps to form an organic–inorganic composite structure that enhances interfacial bonding. At the same time, and more importantly, EVA can form a film in the cement matrix, effectively bridging microcracks and dispersing stress. This mechanism, dominated by physical adsorption and film formation, significantly improves the toughness and impermeability of the material under the premise of not completely sacrificing strength. This mechanism enhances both toughness and impermeability without entirely compromising strength. Multiple systematic experimental studies have provided empirical support for its effectiveness: Kim et al. [17] found that incorporating 3–10% EVA significantly enhanced the flexural strength, toughness, and impact resistance of cement mortar, which was attributed to the bridging effect of the continuous polymer film on microcracks and the enhancement of matrix cohesion; Cai et al. [18] demonstrated the high-temperature resistance of EVA-modified mortar. Specimens with 10% EVA retained approximately 92% of their compressive strength after exposure to 450 °C, benefiting from the protective polymer network formed. Zhang et al. [19] revealed that EVA effectively reduced porosity and optimized pore structure—particularly by decreasing harmful pores larger than 200 nm—thereby enhancing freeze–thaw resistance by 30% and reducing drying shrinkage by 25%. In addition to the aforementioned specific improvements, studies have consistently shown that EVA enhances flexibility and resistance to the environmental degradation of cement-based composites [20]. From the perspective of chemical mechanisms, some studies have suggested that EVA can react with cement hydration products to form calcium acetate and promote the formation of ettringite and C–S–H gel, resulting in densification of the microstructure [21]. However, under typical early hydration conditions, the role of EVA may be mainly based on physical adsorption and space barriers. However, it should be noted that excessive EVA (>12%) will hinder cement hydration due to its air-entraining effect, which eventually leads to a decrease in compressive strength. [22] Although existing studies have confirmed the performance improvements of EVA on cement mortar and concrete, most have focused on macroscopic mechanical properties. There remains a notable lack of systematic research on EVA-modified cement grouting materials, which emphasize specific requirements such as fluidity and stability. In particular, the quantitative impact of EVA dosage on the hydration process, the evolution of the microstructure, and, most critically, the applicability of constitutive models under complex rheological behavior have not been thoroughly investigated. This knowledge gap directly hinders the precise design and efficient application of EVA-modified grouting materials.

In the study of EVA-modified cement-based grouting materials, the accurate characterization of its rheological behavior is very important for the design and engineering application of materials. For a long time, the classical Bingham model has been widely used to describe the rheological behavior of cement pastes based on its advantages of a simple form and clear physical meaning of parameters (yield stress, plastic viscosity). However, the model is essentially linear and has limited predictive ability for common nonlinear rheological properties (such as shear thinning) of polymer-modified systems. In the past decade, the Vipulanandan rheological model [23,24] has shown its superiority over the Bingham model in many research fields. By introducing nonlinear terms, it can more flexibly and accurately fit the rheological curves of complex fluids (such as cement slurry modified by nanomaterials or polymers), and can determine the ultimate shear stress of the fluid. Although it has been confirmed that the incorporation of EVA will significantly change the internal interactions of the slurry, which may produce a large amount of gel-like substances and cause non-Newtonian fluid behavior, the application of this advanced model system to analyze the rheological behavior of EVA-modified cement grouting materials is still rare in domestic and foreign studies. Specifically, for the change in rheological mode from linear to nonlinear under different EVA content, whether the classical Bingham model is still applicable, what the applicable boundary is, and what the differences in fitting accuracy are with the Vipulanandan model are problems that have not been clearly explained. It is of great theoretical value to clarify the applicable boundary and accuracy difference in these two models for the EVA-modified system and to establish an accurate material performance prediction model and guide engineering practice.

Based on the above research background and current status analysis, this study aims to systematically address the existing research gaps by focusing on EVA-modified cement grouting materials through the following innovative work:

Through systematic experimentation, the influence of different P/C ratios (0–20%) of EVA on key properties of grouting materials (fluidity, bleeding rate, and compressive strength at various ages) will be quantitatively investigated to determine the most favorable dosage range. By comprehensively utilizing micro-testing techniques such as SEM, XRD, and FT-IR, the physical and chemical mechanisms of EVA during cement hydration will be revealed, elucidating the micro-level essence behind macro-performance changes. Emphasis will be placed on comparing and analyzing the goodness-of-fit of the Bingham model and the Vipulanandan rheological model for EVA-modified slurries, validating the advanced nature and reliability of the Vipulanandan model in describing this nonlinear system. This will establish a new methodology for the precise characterization of the rheological properties of materials. While significant progress has been made in existing studies, a systematic understanding of how EVA content quantitatively influences the rheological behavior, mechanical properties, and microstructural evolution of cement-based grouting materials remains incomplete. In particular, a comprehensive theoretical framework linking composition structure, processing properties, and final performance is lacking, underscoring the need for multidimensional research that integrates these aspects. This study provides a solid theoretical and experimental foundation for the application of EVA in cementitious grouts, advancing polymer-modified cement research toward greater refinement. It also introduces advanced rheological models, offering new insights and technical support for designing high-performance, multifunctional, and intelligent grouting materials.

## 2. Raw Materials and Experimental Methods

The cement used in this experiment was P·O32.5 grade ordinary Portland cement, and its technical indicators and chemical composition are shown in Table 1.

**Figure 1:**
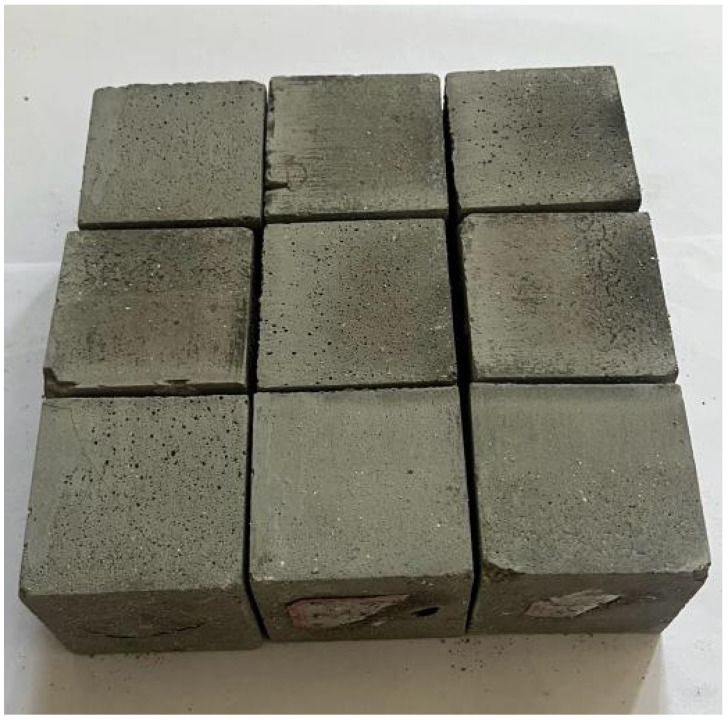
Sample-preparation molding diagram.

**Figure 2:**
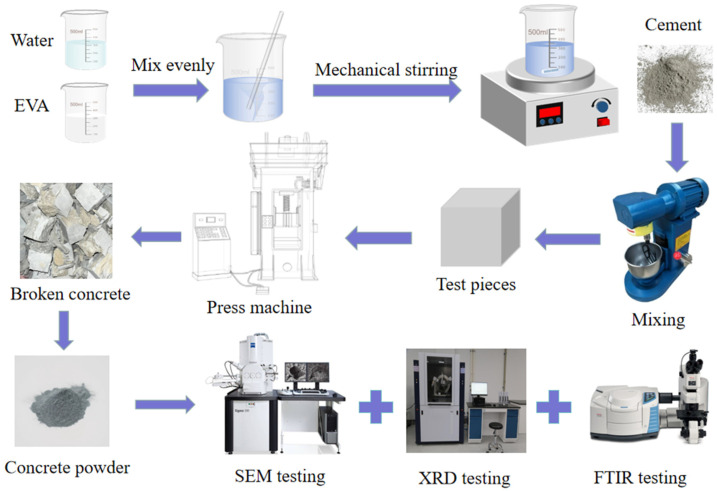
Sample preparation and experimental flowchart.

Materials were weighed according to the proportions in Table 2 and mixed uniformly in a cement mixing pot for three minutes. The mixture was then cast into 40 mm × 40 mm × 40 mm cubic specimens. Nine specimens were prepared for each mix group. After demolding at 1 day, the specimens were placed in a concrete standard curing room. The nine specimens per group were divided into three subsets and cured for 3, 7, and 28 days, respectively. After curing (see Figure 1), compressive strength, microstructure, and other properties of the specimens were tested. The sample preparation and experimental procedure are illustrated in Figure 2.

## 3. Main Testing Methods

### 3.1. Fluidity

**Figure 3:**
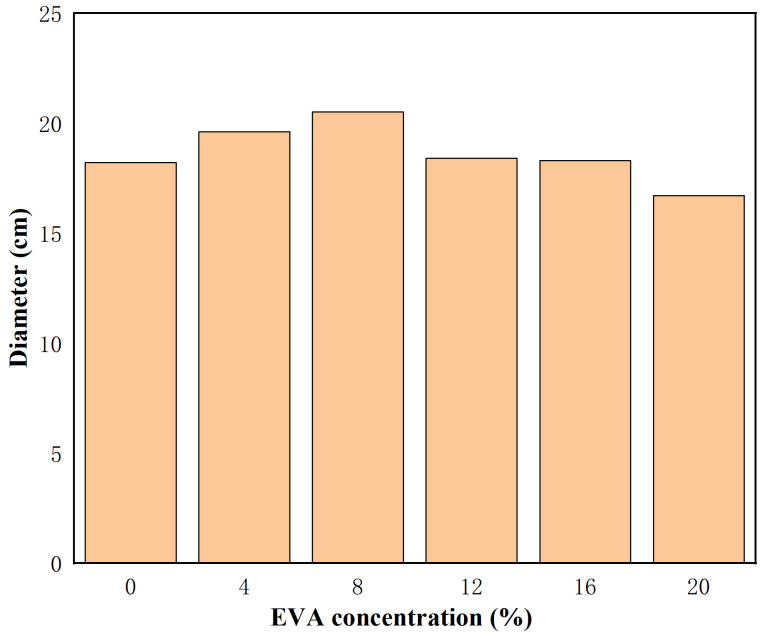
Liquidity results.

The test method and steps of this experiment refer to GB/T 8077-2012. According to the provisions of GB/T 8077, the fluidity test uses a metal, truncated-cone, circular mold with smooth inner walls, an upper diameter of 3.6 cm, a lower diameter of 6.0 cm, and a height of 6.0 cm. During the test, a square acrylic sheet with dimensions of 500 mm × 500 mm × 500 mm is placed in a horizontal position, and a bubble level is placed on the acrylic sheet to assist in leveling. The acrylic sheet and truncated-cone circular mold are wiped with a wet tissue to ensure that their surfaces are moist but without obvious water stains. After the cement injection is evenly mixed, quickly inject the slurry into the truncated-cone mold and wipe off any excess slurry. Lift the truncated-cone mold vertically to allow the cement slurry to flow freely. Measure the diffusion distance of the slurry in two vertical directions after 30 s, and record the average value as the test result. Each group was tested at least three times to take the average value, excluding irregular flow groups. The experimental results are shown in Figure 3.

As shown in Figure 3, the initial fluidity of the fresh slurry increased significantly from 18.2 cm to 20.5 cm as the EVA content rose from 0% to 8%, demonstrating that EVA markedly enhanced slurry flowability. However, when the dosage reached 12% or higher, the fluidity was similar to that of the untreated mix. Analysis of the experimental results indicates that the fluidity showed an initial increase followed by a decrease as EVA content varied, achieving an optimal value at 8% EVA, which represented a 12.6% improvement over the control group. The addition of polymer effectively improved the slurry’s rheological performance.

This phenomenon results from the interaction of multiple competing mechanisms. At low dosages, EVA molecules fully adsorb onto cement particle surfaces. This adsorption disrupts the van der Waals forces between particles through a steric hindrance effect, preventing the formation of flocculated structures and releasing trapped free water. Furthermore, the flexible chains of EVA molecules form a sliding layer between particles, reducing shear resistance, which manifests as decreased plastic viscosity and increased fluidity. When the EVA content exceeds a critical value, the adsorption sites on the cement particle surfaces become saturated. Excess EVA molecules remain free in the solution, where they can form a three-dimensional network through molecular chain entanglement, bridging together multiple particles and increasing the internal friction within the system. At this stage, the “thickening effect” dominates the “dispersion effect,” causing the slurry’s rheological behavior to shift from shear thinning to shear thickening, leading to a consequent loss of fluidity.

### 3.2. Rheological Properties

To investigate the effect of EVA content on the rheological properties of cement grout, a twelve-speed rotational viscometer was used to test the rheological curve of the slurry. This rotational viscometer model has twelve shear strain rates to choose from within the speed range of 1–600 rpm. During the rheological curve testing of the slurry, all rheological tests should be conducted within 10 min of mixing the cement slurry. Increase the shear strain rate from 1022 s^−1^ to 0 s^−1^ within 120 s and record the shear stress corresponding to the shear strain rate. Draw the rheological curve of the modified composite grouting material based on multi-point measurement data to determine the flow pattern of the grouting material during the flow process [25,26,27].

According to the literature, freshly mixed cement-based grouts are typically non-Newtonian fluids, and their rheological curves are generally described by the Bingham model (Equation (1)) [28,29]. The Bingham model offers the advantage of simplicity, allowing yield stress and plastic viscosity to be obtained through linear fitting. While effective for most cement slurries, its primary limitation is its inability to accurately predict the rheological behavior of fluids that deviate from ideal plasticity, especially when nonlinear phenomena like shear thickening or shear thinning occur.

In this test, EVA-modified cement grout may generate gel-like substances in the suspension, altering the interactions between cement particles. The continued applicability of the Bingham model to this system requires verification. Mohammed et al. [30] successfully applied the Vipulananda rheological model (Equation (2)) to simulate nano-silica- and PCE-modified cement slurries. This model can effectively fit nonlinear fluids in complex modified systems and determine the fluid’s ultimate shear stress—a parameter the Bingham model cannot provide. Therefore, the rheological curves of EVA-modified cement grouts in this study were fitted using both the Bingham model and the Vipulananda rheological model.

(1) $$\tau =\tau_{01}+\eta \nu$$

(2) $$\tau -\tau_{02}=\frac{\nu }{A+B\;*\;\nu }$$

Equations (1) and (2): τ is the shear stress (Pa), ν is the shear strain rate (Pa), η is the Bingham plastic viscosity (Pa · s), τ_01_ is the Bingham yield stress (Pa), τ_02_ is the Vipulananda yield stress (Pa), and A and B are model variables.

The various parameters in the fluid model, along with the correlation coefficients, are presented in Table 3 and Table 4.

**Figure 4:**
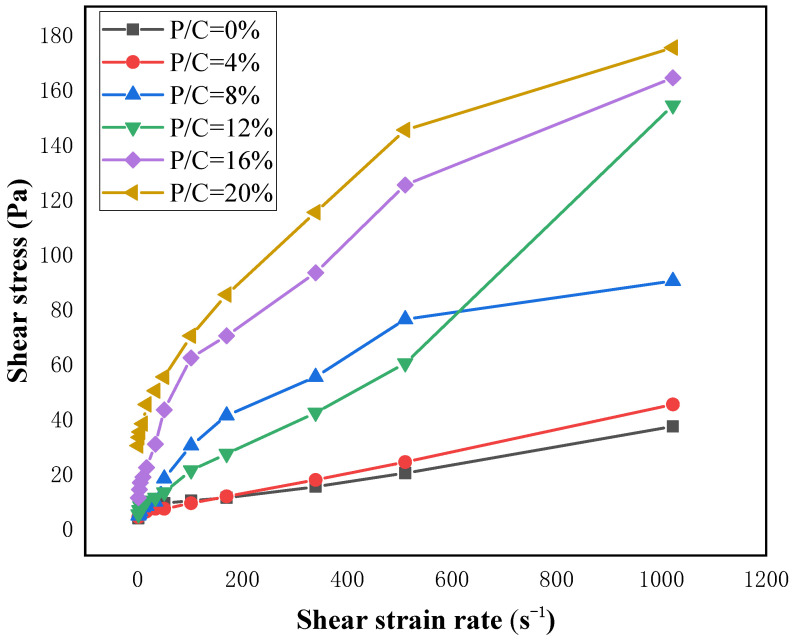
Rheological curves of different concentrations of EVA.

According to the rheological curves of different concentrations of EVA in Figure 4, it can be seen that as the concentration ratio of EVA increases, the shear stress corresponding to each shear strain rate also increases continuously. Therefore, it can be concluded that the addition of different EVA contents significantly changes the properties of the slurry. Additionally, nonlinear fitting was performed on the shear stress corresponding to different concentration ratios in the Bingham model and Vipulananda model. The results are shown in Figure 7, in which the fitting curve of the Vipulanandan model is highly consistent with the experimental data points and its determination coefficient (R^2^) is generally higher than that of the Bingham model (Table 4), especially when the EVA content is high (≥8%). This indicates that the Vipulanandan model can more accurately capture the nonlinear rheological characteristics of EVA-modified slurry, verifying the positive correlation between different concentration ratios and shear stresses.

**Figure 5:**
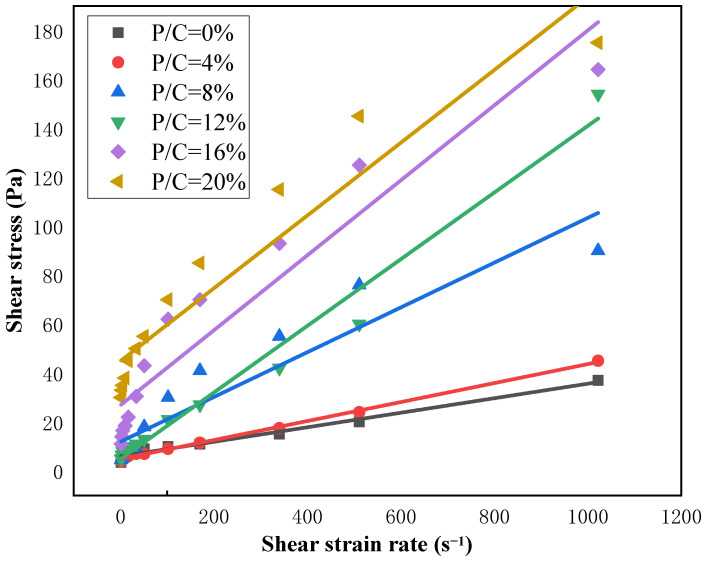
Bingham rheological model fitting modified composite grouting rheological curve.

According to the fitting results of Figure 4 and Figure 5, it can be seen that both models have good fitting effects when the EVA content in the polymer used as a modifier is low. After the increase in the specific gravity of the curing agent in the polymer, there was a significant shear-thinning phenomenon in the modified composite grouting. At this time, the linear relationship between the shear strain rate and shear stress of the fluid weakened, and the fitting accuracy of the Bingham model decreased. However, the Vipulanandan model still exhibited the non-Newtonian characteristics of the fluid well [31].

The fitting results presented in Table 3 and Table 4 reveal the variation patterns of the respective parameters, thereby validating the accuracy of the rheological curve fitting for the modified composite grout. The flow pattern of EVA-modified cement grouting is closely related to its ratio. When the content of EVA is greater than or equal to 8%, there is a significant nonlinear relationship between shear stress and shear strain rate. Removing the irregular 12% ratio group, as the ratio increases, the shear stress and plastic viscosity of the Bingham model and Vipulananda model also show an overall positive correlation. As the polymer concentration increases, the reduced distance between molecular chains significantly enhances the slurry’s adhesive strength. This, in turn, leads to a corresponding rise in both shear stress and plastic viscosity.

**Figure 6:**
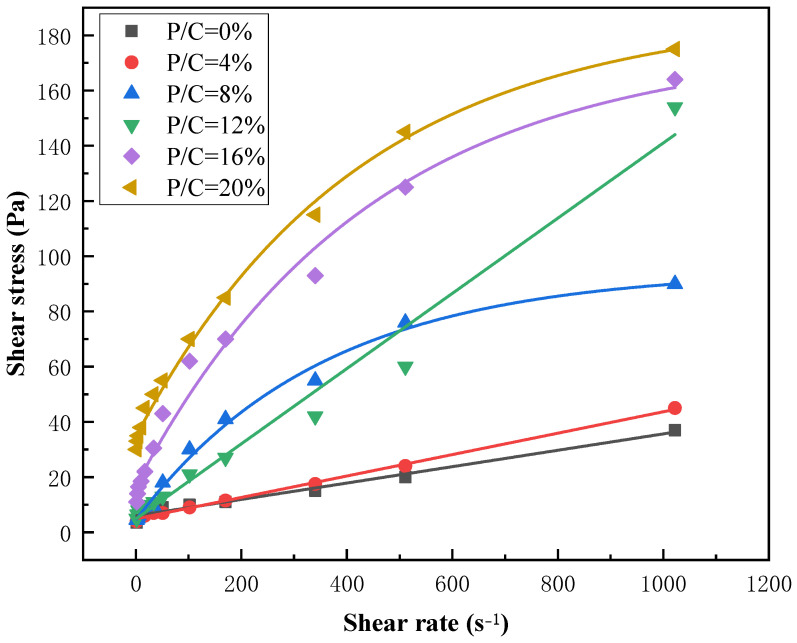
Fitting the rheological curve of modified composite grouting with the Vipulananda rheological model.

When non-Newtonian behaviors such as shear thinning occur in modified composite grouts, the Vipulananda model provides a better fit than the Bingham model. For instance, with an EVA content of 8%, the average root mean square error (RMSE) of the Bingham model fit is 0.868, which is considerably lower than 0.992 achieved by the Vipulananda model, as shown in Table 3 and Table 4. This difference is also visually evident in Figure 5 and Figure 6, where the Vipulananda model’s fitting curve aligns significantly more closely with the measured data points, especially as the EVA ratio increases. Composite slurries typically exhibit high plastic viscosities under low flow rates or static conditions. As the fluid’s shear rate rises, shear stresses between laminar flows cause molecular and particulate structures within the slurry to break down, leading to a macroscopic shear-thinning effect.

**Figure 7:**
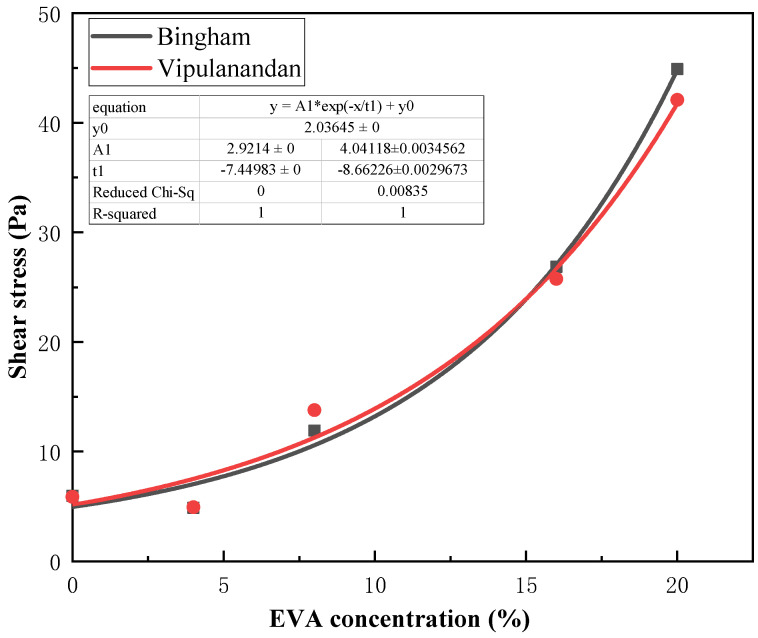
EVA concentration and shear stress fitting curve.

### 3.3. Bleeding Rate

The bleeding phenomenon in cement-based materials refers to the phenomenon of solid particles sinking and water floating in the cement slurry, which is an important indicator for evaluating the stability of the slurry. During testing, less than 100 mL of freshly mixed composite slurry is injected into a 100 mL graduated cylinder, and the cylinder mouth is sealed with plastic wrap. The graduated cylinder is then placed on the test bench. As the test progresses, the solid particles in the slurry begin to sink, and the volume of the upper layer of water gradually increases. At least three parallel specimens were prepared and tested in each group, and the results were averaged. Read and record the volume of upper water every 30 min until it stops increasing after approximately 6 h. The bleeding rate can be calculated using the following formula:

(3) $$B\;=\;\frac{V-V_{0}}{V}$$

Equation (3): B = bleeding rate, V = total volume of slurry test (mL), V_0_ =  volume of solid part in the slurry (mL).

**Figure 8:**
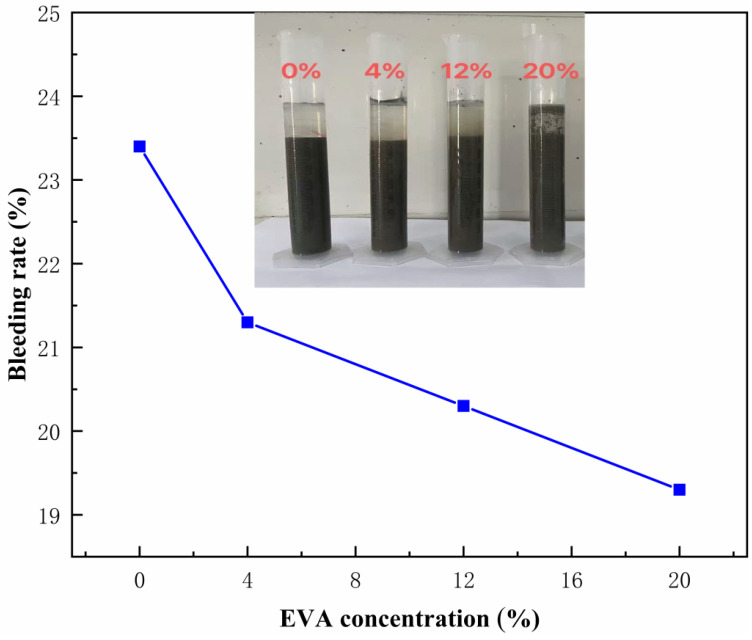
Leakage rate of different EVA concentrations.

From the results in Figure 8, it can be seen that as the proportion of polymer in the concrete slurry gradually increases, the bleeding rate of the concrete slurry shows a significant decreasing trend. The test results show that when the polymer ratio is 4%, the bleeding rate of the concrete slurry is 21.3%. As the polymer ratio gradually increases to 20%, the bleeding rate of the concrete slurry significantly decreases to 19.4%. Further analysis revealed that polymers can form a network structure within the concrete slurry, effectively adsorbing and fixing moisture and reducing the precipitation of free water. At the same time, the interaction between polymers and cement particles enhances the bonding force between cement particles, making the concrete slurry more stable and reducing the bleeding rate. With the increase in polymer ratio, the pore structure within the concrete slurry is optimized and the porosity decreases, which further explains the reason for the decrease in bleeding rate [32].

### 3.4. Analysis of SEM (Scanning Electron Microscopy) Results

**Figure 9:**
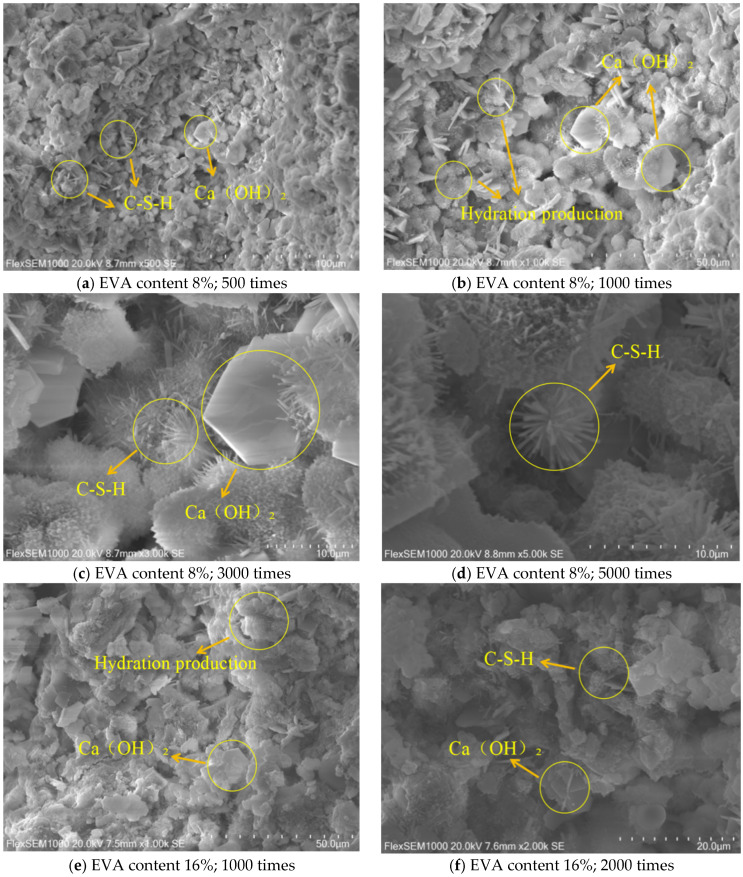

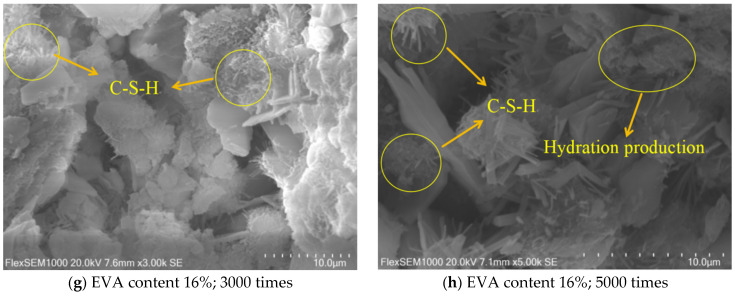
Representative SEM images of asbestiform minerals.

After the compression test of EVA-modified cement grout is completed, approximately 10 mm × 10 mm fragments are taken and soaked in anhydrous ethanol. After soaking for 24 h, the fragments were taken out and placed in a vacuum machine. Vacuum was extracted, and the fragments were sprayed with gold before the experiment. A Flex-SEM1000 scanning electron microscope was used for interface analysis [33,34]. The results are shown in Figure 9. The presence of inherent microcracks in ordinary cement grouting materials leads to a porous microstructure. Adding EVA mitigates this issue, as its smaller particles act as a filler to seal these cracks, significantly improving the compactness of the cement slurry. When added to the cement slurry, they can penetrate the cracks of the cement slurry, making it more compact. This filling effect makes the structure of the cement slurry more compact, thereby reducing the presence of microcracks.

Figure 9 shows that a systematic microstructural study reveals at least four distinct fibrous particle morphologies (including columnar, rod-shaped, tubular, and curled thin-sheet forms). In the EVA-modified grout, the calcium hydroxide hydration products exhibit a clear polymer film coating on their surfaces. This indicates that overly high EVA polymer content can inhibit the formation of C–S–H during hydration, thereby reducing the overall slurry strength. However, the presence of EVA also acts as a structural bridge between cement particles, reinforcing the crystal framework and enhancing the toughness of the cement matrix [35,36,37].

Based on Figure 9a,b,e,f, a substantial amount of C–S–H gel and a small quantity of calcium hydroxide are observed. Furthermore, the high-magnification SEM images in Figure 9c,d,g,h indicate that C–S–H constitutes approximately 50–60% of the hydration products. This ensures the strength of the concrete while enhancing its internal micro-compactness. The fibrous network structure of C–S–H fills the micro gaps between cement particles and strengthens the crystal structure. Accounting for 10–20% of the hydration products, calcium hydroxide is predominantly composed of regular hexagonal platelets. The presence of crystals with non-typical morphologies can be verified by corresponding characteristic peaks in XRD patterns. Calcium hydroxide is encapsulated in a large amount of C–S–H gel, the main product of cement hydration, which has good bonding properties and can significantly enhance the interfacial bonding between calcium hydroxide and the surrounding cement matrix, thereby improving the overall strength and durability of the material. Calcium hydroxide is prone to carbonation reactions in cement-based materials, leading to a decrease in material properties. The encapsulation of calcium hydroxide by C–S–H gel shields it from the external environment, improving its stability and extending the material’s service life. Furthermore, this encapsulation can provide additional nucleation sites for the hydration of calcium hydroxide, promoting the formation of more C–S–H gel, which in turn enhances the material’s strength and durability.

### 3.5. XRD Result Analysis

**Figure 10:**
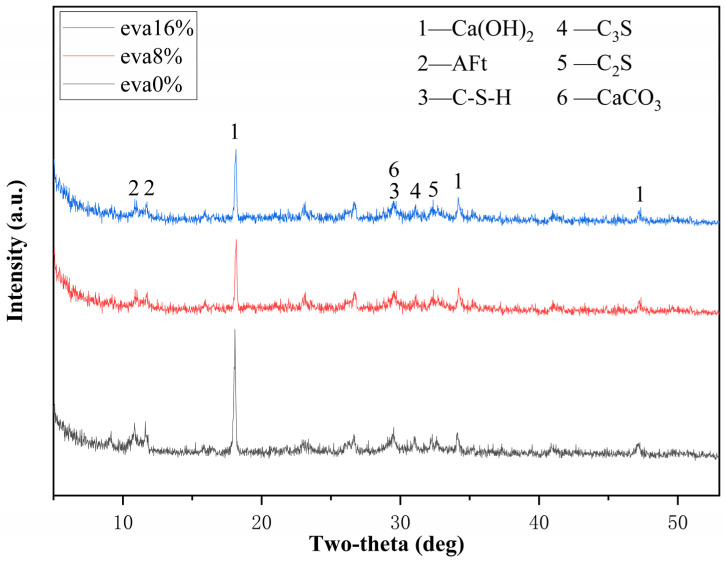
XRD patterns of different EVA contents.

After completing the compressive strength test of EVA-modified grouting material, its fragments were soaked in anhydrous ethanol for 24 h. After soaking for 24 h, the fragments were placed in a vacuum dryer and vacuumed for 12 h. After being vacuumed, the powder was ground and sieved through a 200 mesh sieve. The Rigaku Smartlab X-ray diffractometer was used for testing, and its hydration products were analyzed. Figure 10 presents the XRD patterns of EVA-modified grout with 8% and 16% EVA content, using the 0% EVA sample as the control. The results indicate that the addition of EVA did not alter the types of hydration products formed. The main crystalline hydration product detected is calcium hydroxide (Ca(OH)_2_), along with minor amounts of unhydrated cement. C–S–H, being a gel, does not produce sharp diffraction peaks, and Ca(OH)_2_ is an associated product of the C–S–H formation reaction. Therefore, the formation of Ca(OH)_2_ can be used to infer the progress of C–S–H hydration [38,39,40]. Although the XRD results show no change in the types of hydration products, variations in the intensity of the main Ca(OH)_2_ diffraction peaks for samples with different EVA contents indicate that the rate and extent of hydration product formation are influenced by the EVA content.

At the same age, the diffraction peak of adding 16% EVA is higher than that of adding 8% EVA. This indicates that a small amount of EVA is beneficial for promoting the reaction of C_2_S and C_3_S with water, thereby facilitating the hydration process of C–S–H and improving the strength of the grouting material. When excessive EVA is added, a polymer film will form on the surface of the cement particles and form a whole with the cement grout, thereby inhibiting the hydration of cement and ultimately leading to a decrease in its strength. On the other hand, the flocculent structure formed between EVA and cement particles is beneficial for improving resistance to deformation.

### 3.6. Analysis of FTIR (Fourier Transform Infrared Spectroscopy) Results

After the compressive strength test of the modified slurry of EVA, the crumb is soaked in anhydrous ethanol. After soaking for 24 h, the crumb is placed in a vacuum dryer to be vacuum pumped for 12 h, is vacuum ground into powder and sieved through a 200 mesh sieve, and has its molecular structure characteristics tested and analyzed using a Bruker-D8 Fourier Transformation Infrared Spectrometer [41].

**Figure 11:**
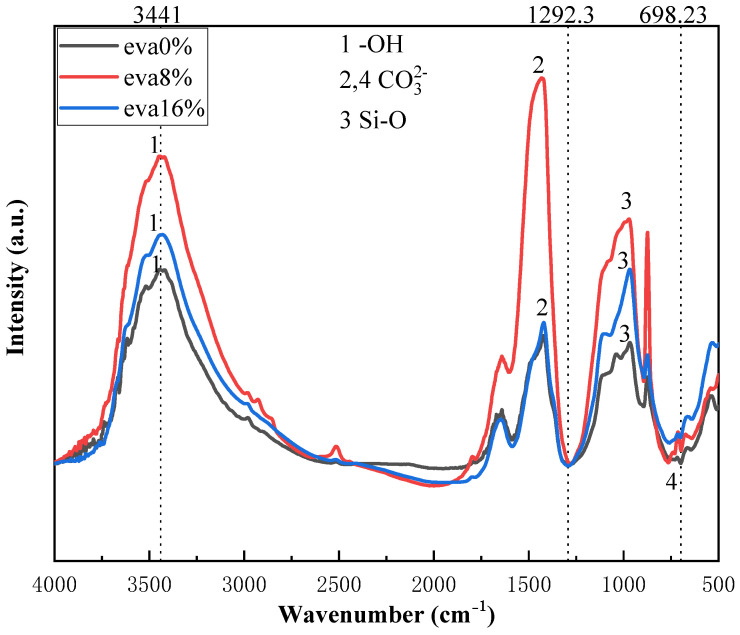
FT-IR spectra in the 4000–500 band.

The red leaf spectrum of EVA-modified cement is shown in Figure 11, and the effect of EVA on cement hydration is reflected by analyzing the functional groups of the modified cement [42,43].

Based on the analysis of the results in Figure 11, characteristic peaks attributed to the -OH bond are observed in the FT-IR spectrum of the modified cement within the 500–4000 cm^−1^ range [44,45]. The -OH bond peak in the 3200–3600 cm^−1^ region corresponds to the formation of calcium hydroxide (CH) and calcium silicate hydrate (C–S–H) gel in the hydration products. The significant enhancement of the -OH peak intensity indicates that the hydration reaction was accelerated, leading to an increased formation of C–S–H gel. Additionally, the intensity of the C–O stretching vibration peak near 1425 cm^−1^ increases noticeably with prolonged carbonation time, suggesting that calcium hydroxide (CH) reacts with CO_2_ in the concrete to form calcium carbonate.

The experimental data reveals an inverse correlation between compressive strength and the peak intensity at 1425 cm^−1^. After 28 days, the 16% EVA sample exhibited a 25% lower compressive strength (12.73 MPa) than the 8% EVA sample (16.98 MPa), in contrast to a 35% increase in the peak intensity at 1425 cm^−1^. This indicates that the formation of calcium carbonate leads to an increase in the porosity of concrete, resulting in a decrease in strength. The FT-IR spectrum shows that the strength of the characteristic peak of the C–S–H gel (970 cm^−1^ Si-O stretching vibration) in the carbonized sample decreases, indicating that the generation of calcium carbonate consumes CH and inhibits the secondary hydration of C–S–H gel. According to the verification of the characteristic peaks of the XRD spectrum (3,6) above, it is consistent with the FTIR results. In the interface reaction of the EVA polymer modifier, the peak intensity of calcium carbonate decreased by 10%, indicating that the polymer film wrapped CH and slowed down the carbonization reaction rate.

### 3.7. Compressive Strength

**Figure 12:**
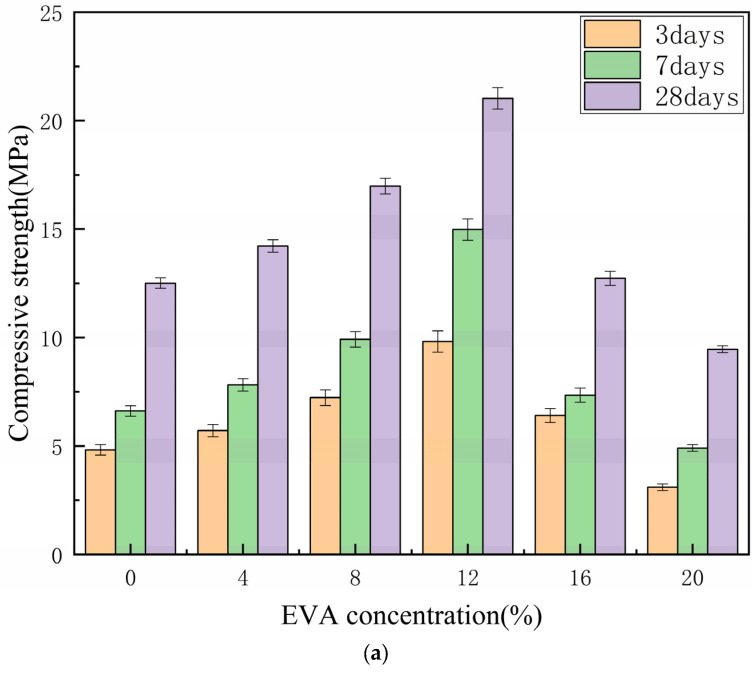

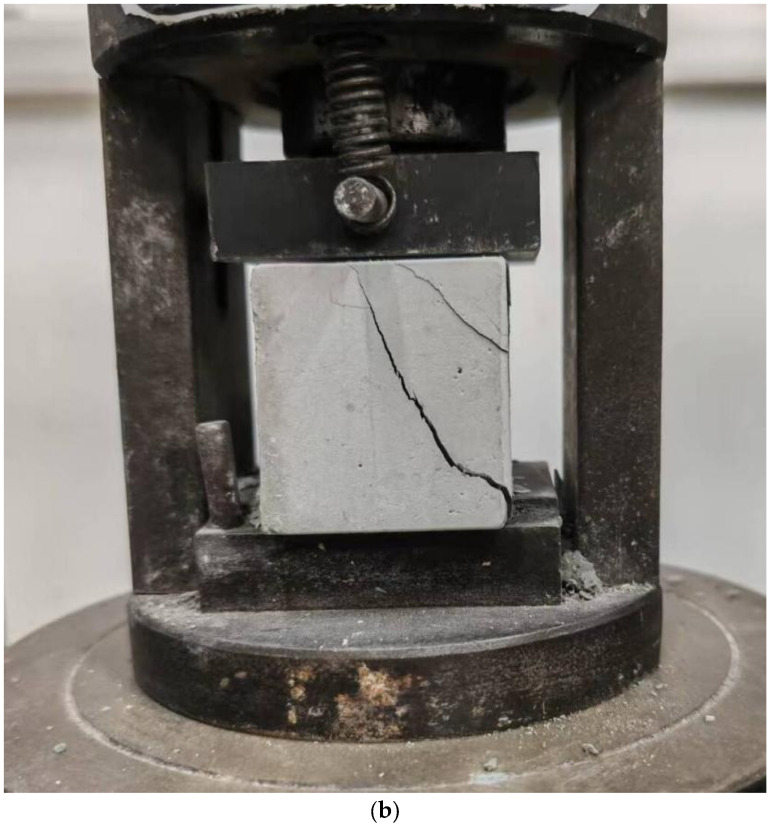
(**a**) Compressive strength diagrams of EVA-modified cement grouting materials with different contents at 3 d, 7 d, and 28 d. (**b**) Failure diagram of standard test block.

According to the curing system of polymer-modified cement slurry, EVA-modified cement grout was cured to the specified age, and then subjected to compressive strength testing. The test results are shown in Figure 12a. The failure diagram of the standard test block is shown in Figure 12b. From Figure 12a, it can be seen that with the increase in EVA content, the compressive strength of the cement grouting material shows a trend of first increasing and then decreasing. When the EVA content reaches 12%, the compressive strength of the test block at 3 d, 7 d, and 28 d reaches the maximum values, which are 9.82 MPa, 14.98 MPa, and 21.03 MPa; compared with the control group, they increased by 120.7%, 124.9%, and 68.1%, respectively. When the content of EVA exceeds 20%, the strength of EVA-modified cement grout is lower than that of the control group. Therefore, the hydrated calcium silicate generated by the hydration reaction is the main source of the early strength of cement. On the one hand, compared with cement particles such as EVA, which have smaller particle sizes, a small amount of EVA can fill the tiny cracks between cement particles, improve the microstructure of cement, and enhance its strength. The reason for the decrease in compressive strength is that as the content of EVA increases, its solubility in water is not significant. After the trial molding, a layer of EVA particles adheres to its surface, and the internal structure of the test block is uneven, resulting in a decrease in its strength. On the other hand, after EVA is added to cement, the moisture content in the grouting material gradually decreases as the hydration reaction progresses. EVA forms a polymer film that adheres to the surface of cement particles and forms a whole with the cement grouting material, thereby inhibiting the hydration of cement and ultimately leading to a decrease in its strength.

### 3.8. Nuclear Magnetic Resonance (NMR) Experiment

**Figure 13:**
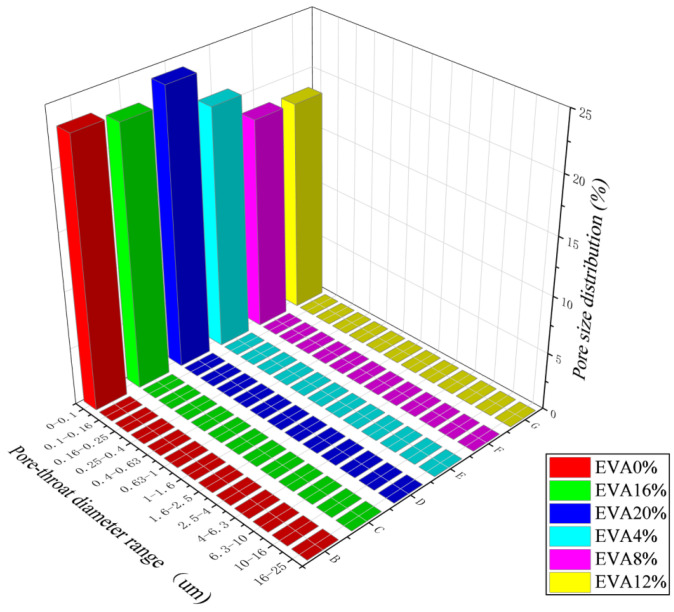
Pore content distribution under different EVA mix proportions.

To verify the relationship between the strength of EVA grout mixtures and their mix proportions, nuclear magnetic resonance (NMR) experiments were conducted, and the microporosity was determined through core analysis. The tests were conducted using a MesoMR12-060-H-1 nuclear magnetic resonance (NMR) analyzer for rock microstructure and moisture dynamics. As shown in Figure 13, the minimum porosity was achieved at an EVA proportion of 12%, indicating favorable hydration reactions between EVA and cement. The hydration products effectively filled the internal pores. By comparing with Figure 12 in Section 3.7, it is evident that the 3-day, 7-day, and 28-day compressive strengths of the 12% EVA mixture all reached their maximum values. When the EVA proportion exceeded 12%, the excessive EVA particles agglomerated, which inhibited the internal hydration process. This resulted in incomplete hydration and led to an increase in internal porosity, consequently reducing the strength.

**Figure 14:**
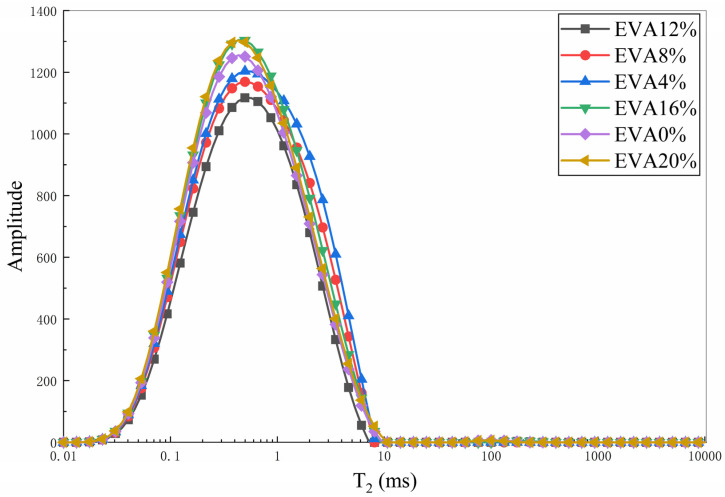
T_2_ spectrum distribution curves under different EVA mix proportions.

The evolution of NMR T_2_ spectra clearly reveals the optimizing effect of EVA on the pore structure of cement paste. As shown in Figure 14, variations in the EVA ratio led to distinct main peak positions in the T_2_ spectra of all specimens, accompanied by a significant reduction in signal amplitude within the large-aperture region (T_2_ > 100 ms), indicating a process of pore refinement and reduction in harmful pores. Compared with the control group, the specimen with 12% EVA exhibited the most pronounced leftward shift in the spectral peak and the greatest reduction in harmful pores, which aligns well with its highest 28-day compressive strength (21.03 MPa). This phenomenon is attributed to the micro-filling effect of EVA particles and the bridging effect of the polymer film formed by EVA on microcracks, collectively promoting the densification of the microstructure.

## 4. Modification Mechanism

Based on the results of a series of physical tests, scanning electron microscopy, X-ray diffraction and Fourier transform infrared spectroscopy, the modification mechanism of EVA on cement grouting materials can be explained. According to research on the modification of other polymers and cement, the interaction between polymers and cement may involve physical adsorption, steric hindrance and potential interfacial chemistry.

**Figure 15:**
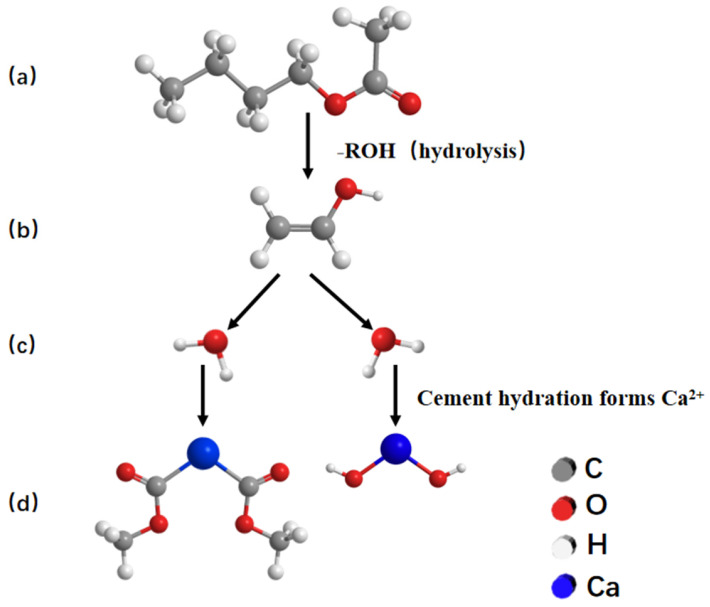
(**a**–**d**) Schematic diagram of the modification mechanism of EVA on cement-based materials.

When EVA is mixed with cement, its molecular chain can be attached to the surface of cement particles by physical adsorption. The modification process is shown in Figure 15. Figure 15a shows a schematic diagram of the interaction between EVA and cement particles. The addition of EVA introduced long molecular chains. In the alkaline environment of cement paste, the acetate group on the EVA chain may be hydrolyzed to a certain extent, exposing polar groups (as shown in Figure 15b). At the same time, the cement particle water releases a large amount of free calcium ions. These calcium ions may have ionic bonding or coordination with polar groups (such as carboxyl groups produced by hydrolysis) on the EVA chain. Due to the presence of a large number of polar groups and free calcium ions in the composite suspension system, they can fill the voids between cement particles and improve the interfacial transition zone between organic and inorganic components in the polymer–cement system. The main interaction between EVA and cement particles is shown in Figure 15c. Finally, the polymer is anchored on the surface of the cement particles through physical winding, adsorption and possible chemical bonding to form a whole, as shown in Figure 15d.

The content of calcium hydroxide in cement hydration products first increases and then decreases with the increase in EVA content. As the content of EVA increases, the number of hydroxyl groups in the solution also increases. Moreover, there is sufficient reaction space on the surface of cement, and the calcium ions on the polymer chains will form nucleation sites for hydroxyapatite.

### Environmental and Health Impacts

While this study focuses on the performance enhancement of cement grouting materials with EVA, it is essential to consider the potential environmental and health impacts when promoting any new modified material. Recent research has highlighted potential health risks associated with the use of nanomaterials, such as nanosilica, and industrial by-products, like fly ash, in civil engineering. For example, due to its ultrafine particle size and high specific surface area, nanosilica may present an inhalation exposure risk during construction if adequate dust control measures are not implemented, potentially affecting the respiratory system.

The EVA material used in this study is inherently a non-toxic, odorless, and environmentally friendly polymer. Once cured, the resulting composite system exhibits a high stability and is unlikely to release harmful substances. However, for future practical engineering applications, it is recommended to conduct systematic life cycle assessments and biosafety testing of EVA and its composite systems to ensure their environmental friendliness and safety throughout their entire life cycle.

## 5. Conclusions

### 5.1. Main Findings

This study systematically investigated the effects of EVA copolymers on the properties of cement-based grouting materials by varying the polymer-to-cement (P/C) ratio from 0% to 20%. The main findings are summarized as follows:

(1) The incorporation of EVA significantly influences the mechanical properties of cement grout. The compressive strength initially increases and then decreases with increasing EVA content. The most favorable dosage was identified as 12%, at which the 28-day compressive strength reached 21.03 MPa—an increase of 68% compared with the plain cement group. Beyond this dosage, excessive EVA impedes hydration and leads to a reduction in strength.

(2) EVA modification alters the microstructure of cement grout by reducing microcracks and enhancing compactness. At appropriate dosages (4–12%), EVA promotes the formation of C–S–H gel and improves the interfacial transition zone. However, at higher contents (>12%), a dense polymer film forms on cement particles, inhibiting further hydration.

(3) The addition of EVA extends the setting time and improves workability. The fluidity of fresh grout initially increases due to the steric hindrance effect and release of free water, peaks at 8% EVA, and decreases thereafter as polymer chain entanglement dominates, increasing viscosity.

(4) Rheological testing demonstrates that the Vipulanandan model fits the behavior of EVA-modified grouts more accurately than the Bingham model, particularly as polymer content increases and pronounced non-Newtonian effects emerge. Both shear stress and plastic viscosity show a consistent increase with higher EVA concentrations.

(5) EVA enhances durability-related properties including impermeability, crack resistance, and toughness through the formation of a flexible polymer network that bridges microcracks and improves stress dispersion.

### 5.2. Limitations of the Study

(1) The study was limited to a 28-day period, lacking long-term performance data.

(2) The effects of environmental variables such as temperature and humidity on the modification efficacy were not considered.

(3) The research primarily focused on mechanical properties and rheological characteristics, without systematically evaluating long-term durability performance.

(4) The EVA content range was set at 0–20%; thus, the effects of higher dosages still require further exploration.

### 5.3. Future Research Directions

(1) Conduct long-term durability studies and investigate performances under different environmental conditions.

(2) Extend the research duration and consider the impact of environmental variables such as temperature and humidity on modification effectiveness.

(3) The developed EVA-modified cement grouting material is particularly suitable for applications such as underground engineering support, rock fracture sealing, and soft ground improvement. Its enhanced strength, toughness, and impermeability contribute to improving the long-term stability and safety of engineering structures.

## Figures and Tables

**Table 1:** Technical indicators and chemical composition of Portland cement.

Density/(kg/m^3^)	Chemical Composition/%					
	CaO	SiO_2_	Al_2_O_3_	MgO	Fe_2_O_3_	SO_3_
3150	63	21.23	6.58	1.6	3.65	2.93

**Table 2:** Sample Preparation Method Table.

Number	Ratio	Cement (g)	Water (g)	EVA (g)
1	W/C 0.8, P/C 0%	100	80	0
2	W/C 0.8, P/C 4%	100	80	4
3	W/C 0.8, P/C 8%	100	80	8
4	W/C 0.8, P/C 12%	100	80	12
5	W/C 0.8, P/C 16%	100	80	16
6	W/C 0.8, P/C 20%	100	80	20

**Table 3:** Fitting parameters and error metrics of the Bingham model for modified composite grouting materials under different polymer ratios.

EVA (%)	Bingham Model $(\tau \;=\;\tau_{01}$+ ην)				
	τ	η	R^2^	RMSE	MAE
0	5.97	0.029	0.977	1.62	0.96
4	4.87	0.038	0.997	1.43	1.05
8	11.91	0.091	0.868	1.56	1.22
12	4.79	0.136	0.976	1.83	0.87
16	26.84	0.153	0.900	1.65	1.58
20	44.89	0.148	0.898	1.49	1.62

**Table 4:** Fitting parameters and error metrics of the Vipulanandan model for modified composite grouting materials under different polymer ratios.

Vipulanandan Model $\left(\tau \;=\;\frac{\nu }{A+B\;*\;\nu }{+\;\tau }_{02}\right)$					
τ	A	B	R^2^	RMSE	MAE
5.88	6.64	0.0046	0.975	1.75	0.96
4.93	4.6	0.0084	0.997	1.45	1.05
13.78	94.99	0.0028	0.992	1.59	1.88
4.38	2.27	0.0059	0.973	1.88	2.17
25.76	176.64	0.0023	0.981	1.67	1.45
42.09	188.32	0.0024	0.994	1.36	1.33

Note: τ is the shear stress (Pa), ν is the shear strain rate ($s^{-1}$), η is the Bingham plastic viscosity (Pa · s), τ_01_ is the Bingham yield stress (Pa), τ_02_ is the Vipulananda yield stress (Pa), and A (Pa · s), and B (Pa) are model variables. RMSE and MAE are both measured in pascals (Pa).

## Data Availability

The original contributions presented in the study are included in the article. Further inquiries can be directed to the corresponding author.
